# Neural reward processing among children with conduct disorder and mild traumatic brain injury in the ABCD study

**DOI:** 10.1017/S0033291725102316

**Published:** 2025-11-04

**Authors:** Hannah Rae Carr, Hedwig Eisenbarth, Dennis Golm, Rebecca Waller, Valerie Brandt

**Affiliations:** 1 Centre for Innovation in Mental Health, University of Southampton, Southampton, UK; 2 School of Psychology, Victoria University of Wellington, New Zealand; 3 Department of Psychology, University of Pennsylvania, Philadelphia, PA, USA; 4 Clinic of Psychiatry, Social Psychiatry and Psychotherapy, Hannover Medical School, Hanover, Germany

**Keywords:** conduct disorder, fMRI, reward, traumatic brain injury

## Abstract

**Background:**

Conduct disorder and childhood head injuries frequently co-occur and are linked to a higher risk of later delinquency. While both are known to disrupt reward-related neural circuits, this study investigated whether their combined presence leads to a unique disruption in these pathways, potentially accounting for the increased risk of delinquency.

**Methods:**

Using neuroimaging data from the baseline (age 9–10) assessment from the Adolescent Brain and Cognitive Development (ABCD) study, four groups were compared: children with conduct disorder (CD, *n* = 588), a mild traumatic brain injury (mTBI, *n* = 1,216), both (mTBI+CD, *n* = 252), and typically developing controls (TD, *n* = 705). Neural activation in eight regions of interest (amygdala, hippocampus, nucleus accumbens, caudal anterior cingulate cortex, rostral anterior cingulate cortex, medial orbitofrontal cortex, thalamus, and insula) during reward anticipation and receipt were assessed during the monetary incentive delay task.

**Results:**

After controlling for several covariates, including sex, ADHD, and internalizing problems, the mTBI+CD group displayed greater left amygdala and hippocampal activation during reward receipt compared to all other groups. While they displayed increased activation in the right hippocampus and thalamus compared to TD controls and the right hippocampus compared to the mTBI group, they did not differ from the CD group. No group differences emerged during reward anticipation.

**Conclusions:**

Increased left amygdala and hippocampus activation in children with conduct disorder and a history of mild traumatic brain injury may reflect robust encoding of emotionally charged reward experiences, potentially reinforcing memory-guided, reward-seeking behaviors.

## Introduction

Conduct disorder (CD) is a prevalent psychiatric disorder associated with numerous maladaptive outcomes (Fairchild et al., 2019), including adolescent delinquency such as criminality, substance use, and antisocial behavior (Hammerton et al., 2019). Interestingly, similar adverse outcomes are associated with a history of childhood mild traumatic brain injury (mTBI) (Mongilio, 2022). Defined as an impact to the head often accompanied by a loss of consciousness or amnesia lasting up to 30 minutes post-injury, this is distinguishable from moderate to severe TBI, which typically involves prolonged post-injury deficits (i.e., a loss of consciousness for greater than 24 hours) and can result in persistent health problems (Mostert et al., 2022). Given that mTBI accounts for roughly 75% of reported head injuries in the US (National Center for Injury Prevention and Control, 2003), continued research is critical to further understand its consequences and long-term effects.

Importantly, CD and childhood mTBI may not be entirely independent constructs. In fact, the literature suggests that CD may increase the risk of childhood mTBI (Vassallo, Proctor-Weber, Lebowitz, Curtiss, & Vanderploeg, 2007) and conversely, mTBI may increase the risk for CD (Delmonico et al., 2024), indicative of a bidirectional relationship (Carr, Hall, Eisenbarth, & Brandt, 2024). Moreover, their co-occurrence has been associated with an increased risk of later maladaptive outcomes, particularly a greater rate of early adolescent delinquency compared to either condition alone (Carr, Hall, & Brandt, 2024). It is therefore paramount that their co-occurrence is explored further, highlighting potential characteristics that may jointly contribute to such a greater risk for delinquency.

One possible explanation for this elevated risk could relate to disruptions to the underlying neural mechanisms of reward processing. The brain’s reward system involves intricate dopaminergic pathways, including those within the mesolimbic (e.g., amygdala, nucleus accumbens [NAc], and hippocampus) and mesocortical systems (e.g., medial orbitofrontal cortex [OFC] and anterior cingulate cortex [ACC]), which receive dopamine inputs from the ventral tegmental area (Cao et al., 2019; Dixon & Dweck, 2022; Rosenberg et al., 2024; Silverman, Jedd, & Luciana, 2015). Although the thalamus and insula are not direct components of this mesocorticolimbic circuit, they are critical for facilitating communication within them (e.g., between the NAc and PFC), and modulate dopamine release from the ventral tegmental area (Haber & Knutson, 2010). Disruptions in these regions during reward processing have, in fact, been linked with both CD (Fairchild et al., 2019; Hawes et al., 2021; Rubia, 2011), mTBI (Cannella, McGary, & Ramirez, 2019; Huang et al., 2020; Mayer, Bellgowan, & Hanlon, 2015) and, consequently, with an increased risk of subsequent antisocial behavior (Hyde, Shaw, & Hariri, 2013; Reyna et al., 2018). As their co-occurrence increases the risk for maladaptive outcomes such as adolescent delinquency above and beyond when they occur in isolation (Carr, Hall, & Brandt, 2024), one may hypothesize that co-occurring CD and childhood mTBI may also be associated with even greater disrupted functioning of reward-related brain regions above and beyond their disrupted functioning in isolation.

However, to date, studies investigating reward-related neural patterns in CD have produced conflicting results (Alegria, Radua, & Rubia, 2016; Noordermeer, Luman, & Oosterlaan, 2016). For example, recent meta-analyses have reported both increased (Noordermeer et al., 2016) and decreased (Alegria et al., 2016) caudate activation during reward processing. These discrepancies likely arise from using different reward-related paradigms, which target different reward mechanisms. That is, some studies have utilized the reward reversal learning task, which focuses on reward learning and cognitive flexibility, while others have used the monetary incentive delay (MID) task, which focuses on reward anticipation, motivation, and response to reward. It is thus essential to recognize the different reward paradigms and the specific reward mechanisms they engage to be consistent with the interpretation of results.

A further critical consideration that must be made is the distinction between reward anticipation and outcome. Reward anticipation refers to incentive motivation - the willingness to expend effort based on learned cues signaling potential reward (Hawes et al., 2021; Swartz et al., 2020). Reward receipt, on the other hand, refers to the hedonic processing related to the outcome of a reward (Hawes et al., 2021). Recent meta-analyses have identified distinct brain regions associated with these phases, including the striatum for reward anticipation and the medial OFC and caudal ACC for reward receipt (Chen, Chaudhary, & Li, 2022; Oldham et al., 2018; Wilson et al., 2018). Subsequently, emerging evidence suggests that CD is uniquely linked to these distinct phases of reward processing (Hawes et al., 2021). For example, a study using baseline data from the Adolescent Brain Cognition Development (ABCD) study at ages 9–10 found that children with Disruptive Behavior Disorders (DBDs, including CD) displayed hypoactivation in the dorsal ACC during reward anticipation and significantly increased activation in cortical (e.g., dorsal ACC) and subcortical (e.g., NAc) regions during reward receipt. These findings stress the need to investigate reward anticipation and receipt independently among children with CD while considering the potential impact of childhood mTBI.

The neural mechanisms underlying reward anticipation and receipt in childhood mTBI remain less well understood. While resting-state fMRI and MEG studies have identified differences in reward-related neural activation in the medial prefrontal cortex, anterior cingulate and anterior insula following a childhood mTBI (Healey, Fang, Smith, Zemek, & Ledoux, 2022; Huang et al., 2020), only one study has directly investigated reward-related activation during a reward-based task (Hogeveen et al., 2024). Using baseline (age 9–10) and 2 year follow-up (age 11–13) data from the ABCD study, this research found hyperactivation of the medial prefrontal and orbitofrontal cortex (OFC) as well as hypoactivation of the ACC and anterior insula during reward anticipation and observed no changes in neural activation during reward receipt (Hogeveen et al., 2024). As this is the first study, to our knowledge, which has investigated the neural mechanisms of reward anticipation and receipt in childhood mTBI, further investigation is needed to clarify the relationship between mTBI and these distinct reward processing phases while also accounting for the influence of CD.

To date, no published studies have investigated the effects of co-occurring CD and childhood mTBI on neural activation during reward anticipation and receipt. Considering their potential risk for co-occurring and a subsequent heightened risk for delinquency, it is paramount that this avenue is explored further. Utilizing baseline data from the ABCD study, the present study aims to identify the neural mechanisms underlying reward processing in children with both CD and mTBI. Specifically, this study aims to identify if disrupted reward-related activation is more pronounced in children with CD and mTBI compared to typically developing youth, and importantly, those with mTBI or CD only. Therefore, our hypothesis was: children with co-occurring CD and mTBI will have a distinct pattern of reward-related neural activation compared to children with mTBI or CD only or TD controls.

## Method

### Participants

Data were obtained from the ABCD study 4.0 data release (https://nda.nih.gov/study.html?id=1299), which recruited 11,874 children aged 9–10 at 21 US research sites using probability sampling (Garavan et al., 2018). Institutional review boards at the 21 participating universities had approved all study procedures, with written assent and parental consent received. This secondary analysis was approved by the University of Southampton Ethics Committee (ID 62100) and adhered to STROBE guidelines.

Exclusion criteria included inadequate MID task performance, indicated by a performance flag set when any trial type yielded fewer than four events with either positive or negative feedback (i.e., too few valid trials in that condition), missing CD and head injury data, and not meeting the criteria for one of four analytical groups (having CD, a mTBI, both, or typically developing, as described below). A breakdown of the exclusion criteria can be seen in Supplementary Figure S1. Most exclusions were due to not meeting group criteria, particularly the stringent standards for the typically developing group, which required no CD, mTBI, or other behavioral or emotional problems in order to minimize potential confounding effects. The final sample consisted of *N* = 2,761 participants (see Table 1 for demographic information of the final sample and further in the Results section).

**Table 1.** Demographic and clinical characteristics by analytical group

	Groups							
	mTBI ± CD (*n* = 252)		CD (*n* = 588)		mTBI (*n* = 1,216)		TD (*n* = 705)	
	M(SD)	n(%)	M(SD)	n(%)	M(SD)	n(%)	M(SD)	n(%)
Demographics								
Age	9.95 (0.61)^a^	—	9.92 (0.63)^a^	—	9.97 (0.62)^a^	—	9.95 (0.62)^a^	—
Male Sex	—	179 (71.0)^a^	—	342 (58.2)^b^	—	680 (55.9)^b^	—	315 (44.7)^c^
Ethnicity	—		—		—		—	
Asian	—	4 (1.6)^a^	—	2 (0.30)^a^	—	18 (1.5)^a^	—	31 (4.4)^b^
Black	—	48 (19.0)^a^	—	168 (28.6)^b^	—	80 (6.6)^c^	—	182 (25.8)^a,b^
Hispanic	—	44 (17.5)^a,b^	—	87 (14.8)^a^	—	198 (16.3)^a,b^	—	143 (20.3)^b^
White	—	114 (45.2)^a^	—	257 (43.7)^a^	—	810 (66.6)^b^	—	290 (41.1)^a^
Other	—	42 (16.7)^a^	—	74 (12.6)^a,b^	—	110 (9.0)^b^	—	59 (8.4)^b^
Conduct Disorder								
CBCL CP Scale (T-score)	68.29 (6.05)^a^	—	67.73 (5.96)^a^	—	50.0 (0)^b^	—	50.0 (0)^b^	—
K-SADS CD Diagnosis	—	103 (40.9)^a^	—	253 (43.0)^a^	—	0^b^	—	0^b^
	—		—		—		—	
TBI	—		—		—		—	
Improbable TBI	—	189 (75.0)^a^	—	0^c^	—	1,001 (82.3)^b^	—	0^c^
Possible mild TBI	—	43 (17.1)^a^	—	0^b^	—	165 (13.6)^a^	—	0^b^
Mild TBI	—	20 (7.9)^a^	—	0^b^	—	50 (4.1)^c^	—	0^b^
Covariates								
Low birth weight	—	55 (21.9)^a,b^	—	135 (23.0)^a^	—	206 (16.9)^b^	—	160 (22.7)^a^
Premature birth	—	46 (18.3)^a^	—	112 (19.0)^a^	—	199 (16.4)^a^	—	111 (15.7)^a^
Smoking	—	83 (32.8)^a^	—	186 (31.7)^a^	—	134 (11.0)^b^	—	53 (7.5)^b^
Alcohol	—	95 (37.6)^a^	—	175 (29.7)^a^	—	360 (29.6)^a^	—	103 (14.6)^b^
ADHD (CBCL T-score)	63.09 (8.97)^a^	—	60.97 (8.20)^b^	—	51.67 (3.72)^c^	—	50 (0)^d^	—
Internalizing problems (CBCL T-score)	61.28 (10.22)^a^	—	58.12 (10.77)^b^	—	46.92 (9.46)^c^	—	33.97 (1.73)^d^	—
Low parental education	—	38 (15.1)^a^	—	140 (23.8)^b^	—	94 (7.7)^c^	—	154 (21.9)^b^
Low household income	—	116 (45.9)^a^	—	318 (54.0)^a^	—	214 (17.6)^b^	—	222 (31.5)^c^
Family conflict	3.08 (2.33)^a^		2.85 (2.16)^a^		1.76 (1.82)^b^		1.77 (1.80)^b^	
MID Performance								
Total earnings ($)	19.90 (13.22)^a^	—	17.71 (17.87)^b^	—	20.77 (13.29)^a^	—	19.76 (15.51)^a,b^	—
Mean reaction time (ms)	313.83 (58.79)^a^	—	331.44 (67.83)^b^	—	313.69 (55.77)^a^	—	322.40 (64.64)^a,b^	—

*Notes:* ^a,b,c,d^For each row, non-matching superscript indicates significant differences between groups.

Abbreviations: CBCL, child behavior checklist; CD, conduct disorder only; CP, conduct problem; K-SADS, the Kiddie schedule for affective disorders and schizophrenia; mTBI, mild traumatic brain injury only; mTBI+CD, co-occurring mild traumatic brain injury and conduct disorder; TD, typically developing controls.

### Measures

#### Conduct disorder

CD was assessed using the computerized versions of the Child Behavior Checklist (CBCL), DSM-orientated Conduct Problems Scale (Achenbach & Ruffle, 2000), and The Kiddie Schedule for Affective Disorders and Schizophrenia for school-aged children (K-SADS-PL DSM-5) (Kaufman, Townsend, & Kobak, 2017). The CBCL consists of 17 items rated from 0 (*not true*) to 2 (*very true* or *often true*), producing a T-score ranging from 50 to 100. Scores ≥65 indicate borderline and clinical ranges of CD. K-SADS-PL DSM-5 generates a CD diagnosis based on DSM-5 diagnostic criteria. CD was evident if participants met DSM-5 criteria via K-SADS-PL or had a CBCL T-score ≥ 65; a commonly used cut-off balancing sensitivity and specificity (Cooper, Di Biase, Bei, Quach, & Cropley, 2023; Cordova et al., 2022; Hou, Mortel, Popma, Smit, & van Wingen, 2025; Krol, De Bruyn, Coolen, & van Aarle, 2006).

#### Mild traumatic brain injury

Head injury status was determined by the Modified Ohio State Traumatic Brain Injury (TBI) Screen (short version) (Corrigan & Bogner, 2007). Parents reported if their child had ever sustained a TBI. These were classified as an improbable (without loss of consciousness or memory loss), possible mild (memory loss but no loss of consciousness), mild (loss of consciousness less than 30 minutes), moderate (loss of consciousness from 30 minutes to 24 hours), or a severe TBI (loss of consciousness greater than 24 hours). A binary variable was created (1 = *mTBI*, 0 = *no head injury*) where a mTBI included an improbable to mild TBI (i.e., a TBI with a loss of consciousness <30 minutes). Those with a reported moderate or severe TBI (*n* = 7) were excluded from analysis as the focus of this study was to investigate mTBI, which are more common in the general population and a comparison with the small number of participants with a moderate to severe TBI would not be appropriate due to low statistical power.

#### Group classification

Participants were assigned to one of four groups based on their CD and mTBI history reported at baseline (age 9–10). The CD group consisted of children with a diagnosis or clinical levels of CD (CBCL T-score ≥ 65) but no reported history of mTBI (*n* = 588). The mTBI group consisted of children with a reported history of mTBI but no diagnosis or clinical levels of CD (CBCL T-score = 50, mTBI, *n* = 1,216). The co-occurring group consisted of children with a reported history of mTBI and a diagnosis or clinical levels of CD (mTBI+CD, *n* = 252). Lastly, a group of typically developing (TD) controls was created based on that used in the previous literature (Hawes et al., 2021) and included those with a CBCL T-score = 50 across eight syndromes and six DSM-5 oriented scales and no reported history of sustaining a head injury (TD, *n* = 705).

#### Covariates

Several prenatal, child-level, and family-level covariates were controlled for in the statistical analysis due to their association with CD (Fairchild et al., 2019; Van Adrichem, Huijbregts, Van Der Heijden, Van Goozen, & Swaab, 2020), head injuries (McKinlay et al., 2010), or reward processing (Blair et al., 2022). These included male sex, ethnicity, participant age, ADHD (as measured by the CBCL ADHD DSM-orientated Scale), internalizing problems (as measured by the CBCL Internalizing Syndrome Scale), low birth weight (< 5 lbs), premature birth (< 37 weeks’ gestation), smoking or alcohol consumption during pregnancy, low parental education, low household income (< $50,000), and family conflict. Family conflict was measured by the Youth Family Environment Scale family conflict subscale, which was modified from PhenX (Moos & Moos, 1994). Nine items including “We fight a lot in our family,” were measured on a binary scale (1 = *true*, 0 = *false*) and summed to create a family conflict score (possible range 0–9) with higher scores indicating higher levels of family conflict.

#### Monetary incentive delay task

**Figure 1.**
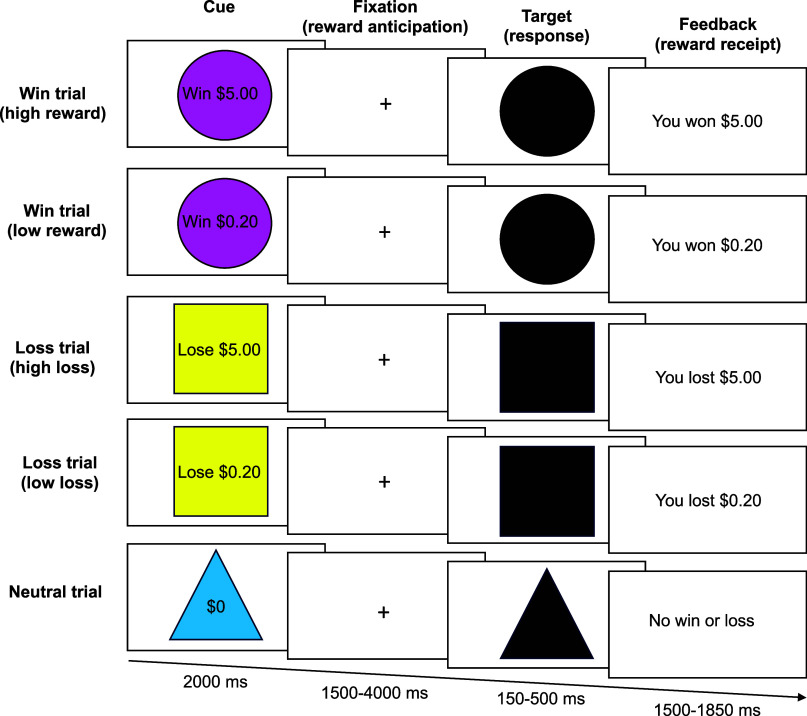
Outline of the monetary incentive delay task for each trial type. This figure highlights the timeline of the monetary incentive delay task as utilized within the ABCD study, broken down by the five trial types.

A version of the MID task (Knutson, Westdorp, Kaiser, & Hommer, 2000) was used to measure neural activation during reward anticipation and reward receipt (see Figure 1) (Casey et al., 2018). The task includes three trial conditions with five trial types: win (+$0.20 or + $5), loss (−$0.20 or -$5), or neutral (+/−$0). Each is associated with a specific incentive cue (a pink circle, yellow square, or blue triangle, respectively). For each trial, participants saw one of these cues on the screen for 2,000 ms, followed by 1,500–4,000 ms of jittered anticipatory delay (a fixation cross). A black target the same shape as the cue then appeared on the screen for 150–500 ms, and participants had to respond as quickly as possible to the target by pressing a button. Successfully pressing the button when the target was on the screen resulted in either winning money (win trial), avoiding losing money (loss trial), or neither winning nor losing money (neutral trial). If participants responded too fast or too slow (i.e., before or after the target appeared on the screen), they did not win money (win trial), they lost money (loss trial), or they neither won nor lost money (neutral trial). This feedback was presented to participants after each trial. To ensure all participants maintained a 60% accuracy rate on this paradigm, the MID task individualized the difficulty by adjusting the target duration based on the overall accuracy rate of the six previous trials. If participants’ accuracy was below 60%, the target duration was increased; if their accuracy was above 60%, the target duration was shortened. Participants completed two runs of the task, each consisting of 50 contiguous trials (20 reward trials, 20 loss trials, and 10 neutral trials) presented in pseudorandom order and lasting approximately 5.5 minutes.

### Image preprocessing

Functional magnetic resonance imaging (fMRI) data from the 21 study sites was harmonized across three 3 T scanner platforms (Siemens Prisma, Philips, General Electric 750) (Casey et al., 2018). Centralized processing, quality control, and analysis of the raw imaging data were performed by the ABCD Data Analysis Informatics and Resource Centre (DAIRC) (Hagler et al., 2019). FreeSurfer v5.3 (Fischl, 2012) was used to create cortical surface reconstruction and subcortical segmentation for the regions of interest. We used beta coefficients available in the ABCD data release (version 4.0) for task-related Blood Oxygen Level Dependent (BOLD) activation during the MID task. These beta coefficients were derived from estimates for the task-related BOLD activation strength computed at the subject level using a general linear model (GLM). They represent the average of the beta coefficients for each of the two MID task runs. We focused on two specific contrasts available in the data release and used in the previous literature (Hawes et al., 2021) (a) reward anticipation: anticipation of large reward ($5) versus no incentive ($0), and (b) reward receipt: positive feedback (won money) versus negative feedback (did not win money). For more information on the preprocessing stage see the supplementary information.

### Regions of interest

**Figure 2.**
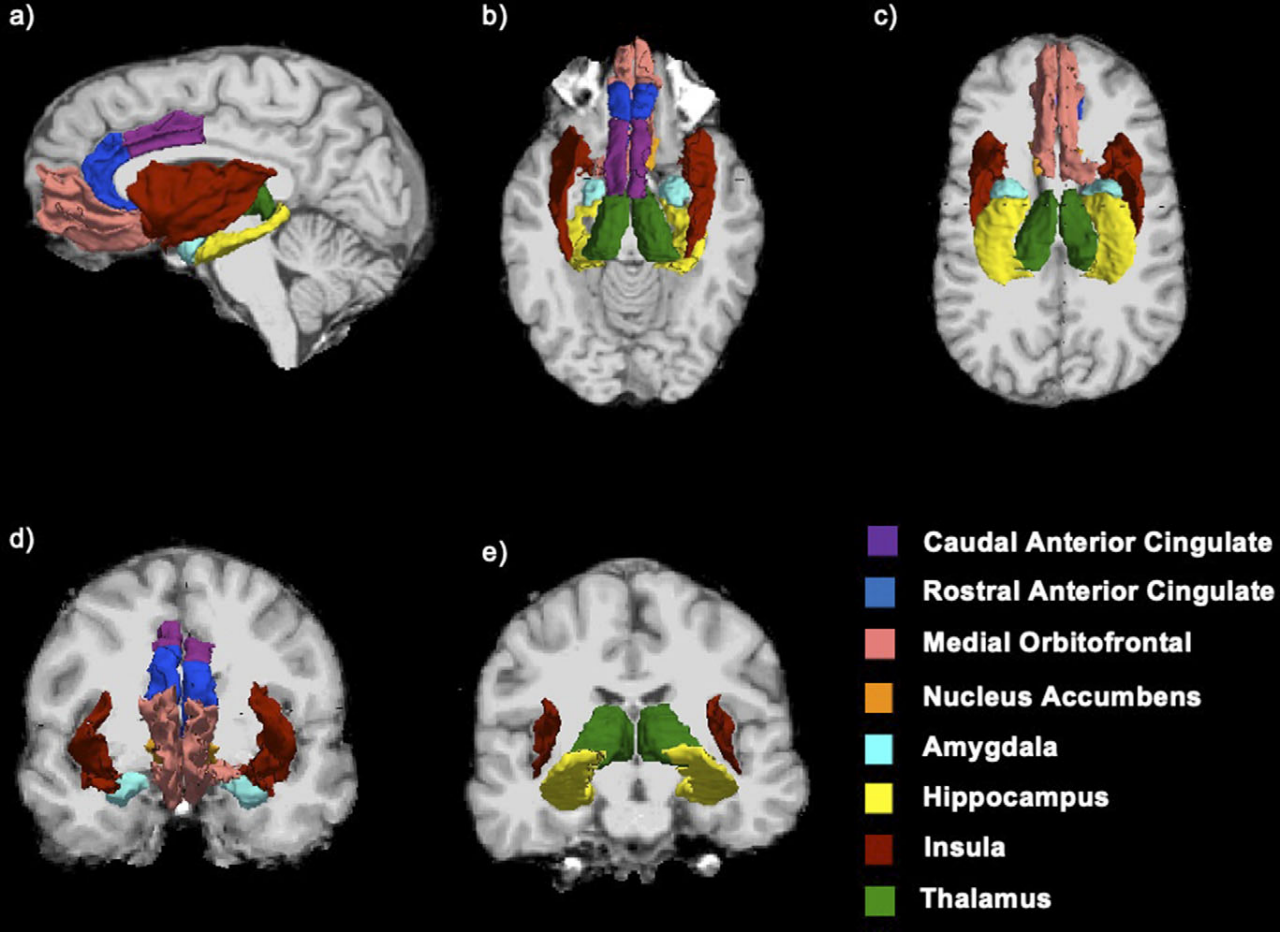
The eight regions of interest used to investigate reward anticipation and reward receipt in children with a history of conduct disorder and/or mild traumatic brain injury. This figure shows the anatomical locations of the eight regions of interest (ROI’s) as viewed from (a) sagittal (left), (b) superior, (c) inferior, (d) anterior, and (e) posterior viewpoints.

Eight ROIs were selected from the brain regions available within the ABCD dataset. These ROIs were selected due to their known associations with reward processing (Cao et al., 2019; Chen et al., 2022; Oldham et al., 2018; Wilson et al., 2018), and either CD or mTBI (Alegria et al., 2016; Cannella et al., 2019; Hawes et al., 2021): amygdala, NAc, hippocampus, medial OFC, caudal ACC, rostral ACC, thalamus, and insula (Figure 2).

### Statistical analysis

Multinomial logistic regression was used to identify activation differences in eight ROIs for reward anticipation and receipt during the MID task (as measured by the beta coefficients) between the following groups: (1) mTBI+CD versus TD controls, (2) CD versus TD controls, (3) mTBI versus TD controls, (4) mTBI+CD versus CD, (5) mTBI+CD versus mTBI, and (6) CD versus mTBI. All models controlled for the following covariates: low birth weight, premature birth, smoking or alcohol consumption during pregnancy, male sex, ethnicity, ADHD, internalizing problems, age, family low SES, and family conflict. Additional models without covariates and with IQ proxies were run (see Supplementary Tables S1–S4), along with sensitivity analyses excluding severe mTBI cases (Supplementary Table S5).

Missing covariate data were handled using maximum likelihood estimation with robust standard errors (MLR), accounting for complex sampling (sibling clustering and site stratification) in Mplus (TYPE = COMPLEX).

The statistical significance for all regression models was set at an alpha level of .05 after applying a false discovery rate (FDR) correction for multiple comparisons using the Benjamini–Hochberg procedure (Benjamini & Hochberg, 1995). Analyses were conducted in Mplus, version 7.4 (Muthén & Muthén, 1998–2017), with FDR-corrections conducted in *R* statistical software version 4.4.1.

## Results

The study included 2,761 children at baseline (1,245 female [45.1%] and 1,471 [53.3%] White). Descriptive statistics, including demographic information, can be seen in Table 1. As expected, there were some significant group differences on most covariates controlled for in the regression models (e.g., a significantly greater proportion of boys in the mTBI+CD group compared to all other groups, see Table 1). Children in the mTBI+CD group reported significantly fewer mild TBIs and greater instances of mTBI’s compared to children in the mTBI group (relevant sensitivity analyses are described below). There were no significant differences in CD (either K-SADS diagnosis or CBCL T-score) between the CD and mTBI+CD groups. Children in the CD group displayed significantly lower total earnings and a slower mean reaction time on the MID task compared to children in the mTBI and mTBI+CD groups.

### Reward anticipation

Multinomial logistic regression models revealed no significant group differences in ROI activation during reward anticipation (Table 2 and Supplementary Figure S2), including in sensitivity analyses (see Supplementary Tables S1 and S3).

**Table 2.** Multinomial regression model results comparing activation during reward anticipation across groups

ROI	Group comparisons					
	CD vs TD	mTBI vs TD	mTBI ± CD vs TD	mTBI ± CD vs CD	mTBI ± CD vs mTBI	CD vs mTBI
	*OR* [95% CI]	*OR* [95% CI]	*OR* [95% CI]	*OR* [95% CI]	*OR* [95% CI]	*OR* [95% CI]
Left hemisphere						
Amygdala	1.11 [0.70, 1.76]	1.35 [0.90, 2.01]	0.97 [0.58, 1.65]	0.88 [0.55, 1.39]	0.72 [0.45, 1.16]	0.83 [0.55, 1.24]
NAc	1.03 [0.67, 1.59]	1.26 [0.90, 1.76]	1.84 [1.05, 3.22]	1.78 [1.06, 2.99]	1.47 [0.87, 2.48]	0.82 [0.57, 1.20]
Caudal ACC	1.26 [0.72, 2.22]	1.52 [0.98, 2.37]	2.30 [1.18, 4.49]	1.82 [1.03, 3.24]	1.51 [0.81, 2.82]	0.83 [0.50, 1.38]
Rostral ACC	1.36 [0.76, 2.44]	1.36 [0.96, 1.93]	2.24 [1.23, 4.07]	1.65 [0.92, 2.95]	1.64 [0.94, 2.85]	1.00 [0.60, 1.66]
Medial OFC	1.00 [0.77, 1.29]	0.98 [0.82, 1.16]	1.38 [0.99, 1.91]	1.38 [1.01, 1.88]	1.41 [1.02, 1.94]	1.02 [0.81, 1.30]
Hippocampus	1.11 [0.57, 2.14]	1.46 [0.85, 2.49]	1.11 [0.49, 2.52]	1.00 [0.47, 2.15]	0.76 [0.36, 1.63]	0.76 [0.42, 1.38]
Thalamus	1.36 [0.70, 2.63]	1.70 [0.99, 2.92]	1.84 [0.81, 4.18]	1.35 [0.63, 2.90]	1.08 [0.50, 2.34]	0.80 [0.44, 1.45]
Insula	1.16 [0.54, 2.50]	1.40 [0.81, 2.44]	1.79 [0.72, 4.47]	1.54 [0.67, 3.55]	1.27 [0.54, 3.02]	0.83 [0.42, 1.65]
Right hemisphere						
Amygdala	1.11 [0.68, 1.82]	1.35 [0.89, 2.03]	1.02 [0.57, 1.83]	0.92 [0.54, 1.57]	0.76 [0.45, 1.29]	0.82 [0.54, 1.27]
NAc	1.01 [0.65, 1.58]	1.14 [0.84, 1.56]	1.73 [0.99, 3.02]	1.71 [1.01, 2.87]	1.51 [0.90, 2.54]	0.89 [0.60, 1.31]
Caudal ACC	1.06 [0.59, 1.92]	1.13 [0.72, 1.77]	1.68 [0.83, 3.39]	1.58 [0.81, 3.07]	1.49 [0.77, 2.88]	0.94 [0.55, 1.61]
Rostral ACC	1.12 [0.66, 1.88]	1.03 [0.70, 1.52]	2.06 [1.08, 3.91]	1.85 [1.00, 3.41]	1.99 [1.09, 3.66]	1.08 [0.68, 1.73]
Medial OFC	0.87 [0.67, 1.12]	0.93 [0.78, 1.12]	1.24 [0.87, 1.77]	1.42 [1.03, 1.98]	1.33 [0.94, 1.88]	0.93 [0.75, 1.17]
Hippocampus	0.92 [0.45, 1.88]	1.59 [0.95, 2.65]	1.09 [0.46, 2.58]	1.19 [0.53, 2.67]	0.69 [0.31, 1.54]	0.58 [0.31, 1.09]
Thalamus	1.24 [0.61, 2.50]	1.62 [0.97, 2.72]	1.86 [0.76, 4.53]	1.50 [0.65, 3.45]	1.15 [0.50, 2.64]	0.76 [0.41, 1.42]
Insula	0.94 [0.47, 1.90]	1.24 [0.74, 2.06]	1.76 [0.74, 4.18]	1.87 [0.84, 4.16]	1.42 [0.63, 3.21]	0.76 [0.41, 1.42]

Abbreviations: ACC, anterior cingulate cortex; CD, conduct disorder only; mTBI, mild traumatic brain injury only; mTBI+CD, co-occurring mild traumatic brain injury and conduct disorder; NAc, nucleus accumbens; OFC, orbitofrontal cortex; OR, odds ratio; TD, typically developing controls.

### Reward receipt

The results from the multinomial logistic regression models for reward receipt are presented in Table 3 and Supplementary Figure S3. Children in the mTBI+CD group showed greater activation of the left amygdala and hippocampus compared to all other groups. While the mTBI+CD group showed differences in activation compared to all other groups in the left insula, right caudal ACC, thalamus, and hippocampus, only activation in the right hippocampus (compared to TD controls and mTBI group) and right thalamus (compared to TD controls) survived the FDR-correction. Further findings, which did not survive the FDR-correction were an increased activation of the bilateral medial OFC, left caudal ACC and right rostral ACC compared to TD controls and the right amygdala compared to mTBI youth as well as greater activation in the left insula and right rostral ACC and medial OFC in the CD group compared to TD controls (Table 3).

**Table 3.** Multinomial regression model results comparing activation during reward receipt across groups

ROI	Group comparisons					
	CD vs TD	mTBI vs TD	mTBI ± CD vs TD	mTBI ± CD vs CD	mTBI ± CD vs mTBI	CD vs mTBI
	*OR* [95% CI]	*OR* [95% CI]	*OR* [95% CI]	*OR* [95% CI]	*OR* [95% CI]	*OR* [95% CI]
Left hemisphere						
Amygdala	1.03 [0.69, 1.54]	0.87 [0.62, 1.20]	2.18* [1.29, 3.66]	2.12* [1.28, 3.49]	2.51* [1.50, 4.21]	1.19 [0.80, 1.77]
NAc	1.22 [0.86, 1.72]	1.07 [0.80, 1.42]	1.51 [0.94, 2.43]	1.24 [0.81, 1.91]	1.42 [0.89, 2.24]	1.14 [0.83, 1.56]
Caudal ACC	1.32 [0.81, 2.15]	1.25 [0.85, 1.85]	2.35 [1.20, 4.62]	1.78 [0.95, 3.34]	1.88 [0.96, 3.69]	1.05 [0.66, 1.68]
Rostral ACC	1.32 [0.87, 2.00]	1.17 [0.83, 1.65]	1.66 [0.99, 2.79]	1.26 [0.79, 2.03]	1.42 [0.87, 2.33]	1.13 [0.76, 1.66]
Medial OFC	1.27 [0.99, 1.62]	1.19 [0.98, 1.43]	1.44 [1.07, 1.93]	1.13 [0.88, 1.46]	1.21 [0.92, 1.59]	1.07 [0.86, 1.33]
Hippocampus	1.66 [0.95, 2.88]	1.12 [0.72, 1.74]	4.20* [2.09, 8.47]	2.54* [1.36, 4.72]	3.76* [1.91, 7.41]	1.48 [0.88, 2.49]
Thalamus	1.27 [0.72, 2.25]	1.13 [0.73, 1.73]	2.14 [0.91, 5.06]	1.68 [0.76, 3.73]	1.90 [0.83, 4.39]	1.13 [0.67, 1.90]
Insula	1.22 [0.68, 2.19]	1.06 [0.66, 1.69]	2.50 [1.17, 5.33]	2.04 [1.00, 4.18]	2.36 [1.15, 4.85]	1.16 [0.68, 1.96]
Right hemisphere						
Amygdala	1.22 [0.83, 1.78]	0.89 [0.64, 1.22]	1.55 [0.96, 2.50]	1.27 [0.80, 2.02]	1.75 [1.06, 2.89]	1.38 [0.93, 2.05]
NAc	1.12 [0.80, 1.58]	0.89 [0.66, 1.20]	1.34 [0.79–2.28]	1.19 [0.74, 1.92]	1.51 [0.89, 2.54]	1.26 [0.90, 1.77]
Caudal ACC	1.27 [0.78, 2.08]	1.09 [0.74, 1.62]	2.32 [1.25–4.32]	1.83 [1.03, 3.26]	2.12 [1.15, 3.90]	1.16 [0.72, 1.86]
Rostral ACC	1.56 [1.03, 2.39]	1.34 [0.95, 1.89]	1.67 [1.01–2.76]	1.07 [0.68, 1.67]	1.25 [0.76, 2.05]	1.17 [0.78, 1.76]
Medial OFC	1.32 [1.02, 1.70]	1.19 [.97, 1.45]	1.47 [1.07, 2.00]	1.11 [0.86, 1.44]	1.24 [0.92, 1.66]	1.11 [0.89, 1.39]
Hippocampus	1.69 [0.95, 3.01]	1.26 [0.78, 2.03]	3.19* [1.65–6.19]	1.89 [1.10, 3.25]	2.53* [1.38, 4.63]	1.34 [0.80, 2.24]
Thalamus	1.43 [0.85, 2.41]	1.35 [0.89, 2.04]	2.98* [1.46–6.07]	2.08 [1.06, 4.08]	2.22 [1.09, 4.49]	1.06 [0.64, 1.76]
Insula	1.11 [0.61, 2.02]	1.09 [0.69, 1.71]	1.60 [0.71–3.59]	1.44 [0.69, 3.04]	1.47 [0.67, 3.22]	1.02 [0.58, 1.77]

Abbreviations: ACC, anterior cingulate cortex; CD, conduct disorder only; mTBI, mild traumatic brain injury only; mTBI+CD, co-occurring mild traumatic brain injury and conduct disorder; NAc, nucleus accumbens; OFC, orbitofrontal cortex; OR, odds ratio; TD, typically developing controls.

* *p* < .05 (FDR-corrected).

Sensitivity analyses excluding participants with more severe mTBIs showed continued elevated activation in the left amygdala and hippocampus in the mTBI+CD group (Supplementary Table S5). Results remained stable when IQ covariates were included (Supplementary Table S4), but no group differences were observed in models without covariates (Supplementary Table S2).

## Discussion

In this study, we investigated whether co-occurring CD and childhood mTBI were associated with greater changes in neural activation during reward processing compared to their effects in isolation (i.e., CD only or a mTBI only). While there were no significant group differences during reward anticipation, significantly higher subcortical neural activation was identified during reward receipt in children with both CD and a history of mTBI.

Consistent with our hypothesis, and expanding upon previous research (Hawes et al., 2021; Oldham et al., 2018), this study identified increased activation in reward-related mesolimbic structures (i.e., left amygdala and hippocampus) during reward receipt in children with CD but only when co-occurring alongside mTBI (mTBI+CD group) compared to all other groups. The amygdala is part of a distributed network of cortical and subcortical regions involved in emotion processing (Lindquist, Wager, Kober, Bliss-Moreau, & Barrett, 2012). Specifically, the left amygdala contributes to evaluating the salience and emotional value of stimuli, including rewards (Costanzo et al., 2015; Šimić et al., 2021). Beyond emotion, the amygdala is also involved in processing uncertainty, detecting threats, regulating arousal, and integrating bodily signals (e.g., interoception). According to the somatic marker hypothesis (Damasio, 1996), it helps associate external stimuli (e.g., reward receipt) with internal emotional and physiological states (or somatic markers) (Šimić et al., 2021). The hippocampus supports this process by encoding the emotional context and somatic markers of reward-related experiences, reinforcing memory for emotionally significant events (Knierim, 2015). Although our data do not directly assess functional interactions, these findings are consistent with prior work suggesting that the hippocampus may integrate emotional information from the amygdala during episodic memory encoding (Shigemune et al., 2010). Thus, this specific pattern of increased activation observed in the hippocampus and the amygdala may indicate that children with co-occurring CD and mTBI likely encode both contextual and emotional aspects of reward events more intensely. Such enhanced encoding may drive future memory-guided reward seeking behaviors in the pursuit of similar rewards. These findings were only identified when CD co-occurred alongside mTBI suggestive of an interplay between the two, which should be considered in future CD research investigating neural activation patterns during reward receipt.

Our findings regarding reward receipt further align with previous mTBI work highlighting no significant activation differences during reward receipt in children with mTBI alone (Hogeveen et al., 2024). However, we expand upon these findings by highlighting increased neural activation during reward receipt in children with mTBI only when co-occurring alongside CD. This thus suggests that heightened neural activity patterns during reward receipt may be specific to a subset of children with co-occurring mTBI and CD. Notably, although the mTBI+CD group had significantly more severe mTBIs than the mTBI-only group, excluding these cases did not change the overall findings. This suggests that the increased activation is specific to children with both mTBI and CD, rather than being driven by injury severity alone. This may thus further highlight why research should consider their association when investigating mTBI. As such, and similarly to CD research, we, thus, recommend further exploration of the role of reward processing in children with a mTBI both when co-occurring alongside CD, or when controlling for the influence of CD.

We found no significant differences in neural activation during reward anticipation across clinical groups. This contrasts with the previous literature in children with DBDs (Hawes et al., 2021) and mTBI (Hogeveen et al., 2024). These discrepancies may stem from differences in sample characteristics. For instance, Hawes et al. (2021) identified significant alterations in reward anticipation in children with DBDs, but their sample included both children with CD and oppositional defiant disorder (Hawes et al., 2021), suggesting that reward anticipation deficits may be more characteristic of other DBDs rather than CD alone. Similarly, our mTBI group included all children at baseline with a history of sustaining any form of mild head injury, from an improbable TBI to a mTBI, whereas Hogeveen et al.’s (2024) focused on those with a mTBI with a loss of consciousness. This suggests that changes to neural activation patterns during reward anticipation may be more pronounced in those with more severe mTBI. Additionally, Hogeveen et al.’s (2024) mTBI sample included children who sustained head injuries between study visits, reducing the delay between sustaining the reported mTBI and completing the reward-based paradigm. The longer delay in our study may have diminished the observable effects of mTBI on neural activation patterns during reward anticipation.

Children with co-occurring mTBI and CD (mTBI+CD group) exhibited moderate increases in neural activation in the right caudal ACC, hippocampus, and thalamus compared to all other groups. However, it must be stressed that not all findings survived the FDR-correction, and these results should therefore be interpreted with caution. These activation patterns were not observed in the CD or mTBI groups compared to TD controls (even prior to FDR-correction) suggesting that these heightened activation patterns may be somewhat unique to the co-occurrence of mTBI and CD compared to children with CD or mTBI alone or typically developing youth. The thalamus plays a crucial role in relaying sensory and emotional information to the striatum (Wolff, Morceau, Folkard, Martin-Cortecero, & Groh, 2021). As such, increased thalamic activation in the mTBI+CD group may reflect heightened emotional and sensory processing of reward-related stimuli. Additionally, the caudal ACC, which integrates inputs from the thalamus as well as the amygdala, and hippocampus, is involved in evaluating whether received rewards meet expectations, monitoring errors, and adjusting motivation (Umemoto, HajiHosseini, Yates, & Holroyd, 2017). Together, the heightened activity in these interconnected regions could suggest a unique neural profile in children with mTBI+CD, characterized by an amplified sensitivity to the emotional and sensory aspects of monetary reward, as well as an increased arousal to reward expectations and motivation. Nonetheless, given that several of the group differences did not survive the FDR-correction, (only those involving the left hippocampus (vs all groups), right hippocampus (vs TD and mTBI groups), and right thalamus (vs. TD control)) our interpretations are exploratory and should be considered hypothesis-generating rather than conclusive. Further research is, thus, necessary to expand upon these findings and hypotheses further.

### Strengths and limitations

We note that the large sample and the analysis design, which controls for ADHD alongside several relevant covariates across the child and family levels are strengths of our research. However, we note several limitations of the current study. A cross-sectional methodology limits us to identifying correlations and not developmental changes. It remains unclear whether the observed reward receipt activation patterns persist, diminish, or intensify over time. Whilst the absence of group differences during reward anticipation may reflect a diminishing impact of early mTBI, we cannot identify whether this could have a similar but delayed impact on reward receipt later in development. Longitudinal research is needed to determine whether these effects are transient or reflect more enduring neurobiological alterations requiring intervention. While no prior study has explored how CD and mTBI jointly affect reward processing over time, behavioral research suggests early head injury may exacerbate the impact of conduct problems on delinquency in early adolescence, though this effect appears to diminish by age 17 (Carr, Hall, & Brandt, 2024). This implies that mTBIs influence may be transient, but further research is needed to confirm this. These findings highlight the need for longitudinal studies to understand how CD and mTBI interact to shape neurodevelopment and behavior. Finally, the MID task restricts our findings to monetary reward. To identify if these neurological pathways are similar across various reward subtypes, a comparable research framework should be applied to other paradigms, for example, social reward. Additionally, given the relevance of punishment sensitivity for CD (Elster et al., 2025), it would be valuable to explore punishment-related processing using tasks specifically designed to assess negative outcomes.

## Conclusions

Overall, we found novel evidence that furthers our understanding of the neural pathways associated with children with co-occurring mTBI and CD. That is, this group was characterized by significantly greater activation in the left amygdala and hippocampus during reward receipt compared to typically developing children and, importantly, children with CD or mTBI alone. The increased amygdala activation may suggest an emotional hyperresponsivity to positive reward outcomes in children with co-occurring CD and mTBI, while the increased hippocampal activation may indicate more robust encoding of such emotionally charged reward experiences, potentially reinforcing memory-guided, reward-seeking behavior. Together, these findings could be an important first step in understanding the stronger drive toward reward-seeking behaviors in this population, which may contribute to the higher risk of maladaptive outcomes, such as delinquency previously observed in those with co-occurring CD and mTBI (Carr, Hall, & Brandt, 2024).

## Supporting information

Carr et al. supplementary material Carr et al. supplementary material

## Data Availability

Data used in the preparation of this article were obtained from the Adolescent Brain Cognitive Development (ABCD) Study (https://abcdstudy.org), held in the NIMH Data Archive (NDA). This is a multisite, longitudinal study designed to recruit more than 10,000 children aged 9–10 and follow them over 10 years into early adulthood. ABCD consortium investigators designed and implemented the study and/or provided data but did not necessarily participate in the analysis or writing of this report. This paper reflects the views of the authors and may not reflect the opinions or views of the NIH or ABCD consortium investigators. The ABCD data repository grows and changes over time. The ABCD data used in this report came from https://doi.org/10.15154/1523041.

## Supplementary material

The supplementary material for this article can be found at http://doi.org/10.1017/S0033291725102316.
