# Supplementary material for: Neural reward processing among children with conduct disorder and mild traumatic brain injury in the ABCD study
Source: Psychol Med. 2025 Nov 4;55:e333. doi: 10.1017/S0033291725102316 (PMC13058627; doi:10.1017/S0033291725102316)
Supplement: Carr et al. supplementary material [file S0033291725102316sup001.docx]

**Neural reward processing among children with conduct disorder and mild traumatic brain injury in the ABCD study**

**Figure S1.** Flowchart of Study Inclusion/Exclusion Criteria

Enrolled in study at baseline (N=11,874)

Flagged MID performance

(N=322)

Missing data (N=166)

Adequate MID performance

(N=11,388)

Missing head injury data (N=2)

Complete head injury data

(N=11,386)

Missing Conduct Disorder data (N=123)

Complete Conduct Disorder data (N=11,263)

Conduct Disorder (N=588)

Mild traumatic brain injury (N=1,216)

Both (N=252)

Typically developing (N=705)

Meet criteria for one of four analytical groups (N=2,761)

**Imaging Preprocessing**

Structural T1-weighted images were first processed to create cortical surface reconstructions and subcortical segmentations for regions of interest (ROIs). Registration between B0 field maps and the structural T1-weighted images was performed using mutual information with coarse pre-alignment to atlas brains, followed by a rigid-body transformation matrix to align the fMRI volumes to the T1-weighted images. All fMRI data remained in native space at 2.4 mm isotropic resolution throughout preprocessing. Initial steps included head motion estimation and correction, image distortion correction (addressing B0 field inhomogeneities and gradient nonlinearities), normalization of voxel time series by dividing by the mean signal over time, and removal of initial frames to allow signal equilibration. Fast oscillatory signals related to respiration (0.31–0.43 Hz) were temporally filtered using an infinite impulse response notch filter, and framewise displacement (FD) was calculated from the filtered motion estimates. Frames with FD > 0.9 mm were censored. Time series were then sampled 1 mm into cortical grey matter using surface reconstructions, smoothed along the cortical surface (5 mm), and mapped to the standard FreeSurfer surface atlas (fsaverage). Nuisance regressors including motion estimates, their derivatives, and polynomial trends were included in the modelling. General linear models (GLMs) were run with AFNI’s 3dDeconvolve, modelling task events using a two-parameter gamma variate basis function plus its temporal derivative (‘SPMG’ option). Beta coefficients and standard errors of the mean (SEM) were computed for each voxel and ROI. These values were averaged across two runs per subject, weighted by degrees of freedom calculated after censoring (frames minus model parameters). Runs with fewer than 50 degrees of freedom were excluded, and contrasts with high SEM (RMS > 5% signal change) were censored and replaced with empty cells to reduce the impact of motion artifacts on group-level analyses.

**Table S1.** Multinomial Regression Model Results Comparing Activation During Reward Anticipation Across Groups With No Covariates

|  | **Group comparisons** | | | | | |
| --- | --- | --- | --- | --- | --- | --- |
|  | **CD vs TD** | **mTBI vs TD** | **mTBI+CD vs TD** | **mTBI+CD vs CD** | **mTBI+CD vs mTBI** | **CD vs mTBI** |
| **ROI** | ***OR* [95% CI]** | ***OR* [95% CI]** | ***OR* [95% CI]** | ***OR* [95% CI]** | ***OR* [95% CI]** | ***OR* [95% CI]** |
| **Left hemisphere** |  |  |  |  |  |  |
| Amygdala | 0.60 - 1.37 | 0.82 - 1.55 | 0.37 - 1.17 | 0.39 - 1.33 | 0.33 - 1.01 | 0.55 - 1.17 |
| NAc | 0.63 - 1.33 | 0.92 - 1.63 | 1.02 - 2.92 | 1.08 - 3.33 | 0.85 - 2.33 | 0.53 - 1.05 |
| Caudal ACC | 0.67 - 1.80 | 0.93 - 1.98 | 1.09 - 4.09 | 0.96 - 3.87 | 0.84 - 2.92 | 0.52 - 1.27 |
| Rostral ACC | 0.79 - 1.92 | 0.92 - 1.73 | 1.09 - 3.18 | 0.84 - 2.74 | 0.89 - 2.44 | 0.65 - 1.46 |
| Medial OFC | 0.81 - 1.26 | 0.85 - 1.21 | 1.00 - 1.86 | 0.99 - 1.85 | 1.01 - 1.80 | 0.83 - 1.20 |
| Hippocampus | 0.57 - 1.90 | 0.80 - 1.99 | 0.48 - 2.46 | 0.43 - 2.48 | 0.39 - 1.88 | 0.48 - 1.43 |
| Thalamus | 0.53 - 1.71 | 0.91 - 2.33 | 0.50 - 2.53 | 0.50 - 2.79 | 0.35 - 1.70 | 0.38 - 1.14 |
| Insula | 0.54 - 1.98 | 0.70 - 1.86 | 0.58 - 3.21 | 0.53 - 3.28 | 0.54 - 2.69 | 0.51 - 1.64 |
|  |  |  |  |  |  |  |
| **Right hemisphere** |  |  |  |  |  |  |
| Amygdala |  |  |  |  |  |  |
| NAc | 0.67 - 1.42 | 0.86 - 1.49 | 1.11 - 3.06 | 1.10 - 3.25 | 1.01 - 2.63 | 0.61 - 1.23 |
| Caudal ACC | 0.55 - 1.58 | 0.71 - 1.58 | 0.76 - 2.96 | 0.76 - 3.37 | 0.74 - 2.69 | 0.54 - 1.43 |
| Rostral ACC | 0.69 - 1.68 | 0.71 - 1.40 | 1.08 - 3.70 | 0.96 - 3.61 | 1.11 - 3.61 | 0.71 - 1.62 |
| Medial OFC | 0.75 - 1.23 | 0.77 - 1.16 | 0.99 - 1.94 | 1.04 - 2.02 | 1.08 - 1.99 | 0.84 - 1.22 |
| Hippocampus | 0.42 - 1.42 | 0.77 - 1.92 | 0.35 - 2.04 | 0.43 - 2.77 | 0.30 - 1.59 | 0.37 - 1.09 |
| Thalamus | 0.53 - 1.78 | 0.90 - 2.28 | 0.57 - 3.18 | 0.56 - 3.42 | 0.41 - 2.13 | 0.39 - 1.17 |
| Insula | 0.50 - 1.65 | 0.67 - 1.69 | 0.69 - 3.70 | 0.73 - 4.28 | 0.68 - 3.33 | 0.49 - 1.47 |

*Note.* CD = conduct disorder only; TD = typically developing controls; mTBI = mild traumatic brain injury only; mTBI+CD = co-occurring mild traumatic brain injury and conduct disorder; OR = odds ratio; NAc = nucleus accumbens; ACC = anterior cingulate cortex; OFC = orbitofrontal cortex.

**Table S2.** Multinomial Regression Model Results Comparing Activation During Reward Receipt Across Groups With No Covariates

|  | **Group comparisons** | | | | | |
| --- | --- | --- | --- | --- | --- | --- |
|  | **CD vs TD** | **mTBI vs TD** | **mTBI+CD vs TD** | **mTBI+CD vs CD** | **mTBI+CD vs mTBI** | **CD vs mTBI** |
| **ROI** | ***OR* [95% CI]** | ***OR* [95% CI]** | ***OR* [95% CI]** | ***OR* [95% CI]** | ***OR* [95% CI]** | ***OR* [95% CI]** |
| **Left hemisphere** |  |  |  |  |  |  |
| Amygdala | 1.13 [0.72, 1.80] | 1.37 [0.91, 2.06] | 1.02 [.60 - 1.73] | 0.90 [0.60 - 1.41] | 0.74 [0.46 - 1.20] | 0.83 [0.55 - 1.25] |
| NAc | 1.10 [0.70, 1.70] | 1.26 [0.90, 1.78] | 1.94 [1.11 - 3.41] | 1.78 [1.06 - 2.96] | 1.54 [0.91 - 2.59] | 0.87 [0.59 - 1.27] |
| Caudal ACC | 1.33 [0.76, 2.35] | 1.51 [0.97, 2.37] | 2.45 [1.26 - 4.79] | 1.84 [1.04 - 3.25] | 1.62 [0.87 - 3.03] | 0.88 [0.53 - 1.47] |
| Rostral ACC | 1.39[0.77, 2.51] | 1.36 [0.96, 1.93] | 2.31 [1.27 - 4.21] | 1.66 [0.93 - 2.98] | 1.69 [0.98 - 2.93] | 1.02 [0.61 - 1.70] |
| Medial OFC | 1.01 [0.78, 1.32] | 0.98 [0.82, 1.16] | 1.39 [1.00 - 1.94] | 1.37 [1.01 - 1.86] | 1.43 [1.04 - 1.95] | 1.04 [0.82 - 1.32] |
| Hippocampus | 1.13 [0.58, 2.18] | 1.47 [0.86, 2.52] | 1.18 [0.52 - 2.69] | 1.05 [0.50 - 2.23] | 0.80 [0.37 - 1.73] | 0.77 [0.42 - 1.39] |
| Thalamus | 1.49 [0.76, 2.93] | 1.72 [0.99, 2.97] | 2.04 [0.90 - 4.65] | 1.37 [0.64 - 2.91] | 1.19 [0.56 - 2.55] | 0.87 [0.48 - 1.58] |
| Insula | 1.24 [0.58, 2.66] | 1.41 [0.81, 2.46] | 1.98 [0.80 - 4.92] | 1.60 [0.70 - 3.65] | 1.40 [0.59 - 3.30] | 0.88 [0.44 - 1.75] |
|  |  |  |  |  |  |  |
| **Right hemisphere** |  |  |  |  |  |  |
| Amygdala | 1.08 [0.66, 1.78] | 1.33 [0.88, 2.02] | 1.03 [0.57 - 1.84] | 0.95 [0.57 - 1.60] | 0.77 [0.46 - 1.31] | 0.81 [0.53 - 1.25] |
| NAc | 1.07 [0.69, 1.66] | 1.14 [0.83, 1.56] | 1.83 [1.05 - 3.19] | 1.71 [1.02 - 2.87] | 1.60 [0.96 - 2.68] | 0.94 [0.64 - 1.38] |
| Caudal ACC | 1.12 [0.62, 2.01] | 1.13 [0.71, 1.78] | 1.79 [0.89 - 3.58] | 1.59 [0.83 - 3.05] | 1.59 [0.83 - 3.03] | 1.00 [0.59 - 1.68] |
| Rostral ACC | 1.16 [0.69, 1.96] | 1.04 [0.70, 1.53] | 2.14 [1.14 - 4.05] | 1.85 [1.01 - 3.39] | 2.07 [1.14 - 3.77] | 1.12 [0.70 - 1.79] |
| Medial OFC | 0.89 [0.69, 1.17] | 0.93 [0.77, 1.12] | 1.27 [0.88 - 1.82] | 1.42 [1.02 - 1.96] | 1.37 [0.97 - 1.92] | 0.97 [0.77 - 1.21] |
| Hippocampus | 0.94 [0.46, 1.92] | 1.59 [0.95, 2.68] | 1.14 [0.48 - 2.69] | 1.21 [0.54 - 2.71] | 0.71 [0.32 - 1.60] | 0.59 [0.31 - 1.10] |
| Thalamus | 1.41 [0.69, 2.85] | 1.63 [0.97, 2.76] | 2.15 [0.88 - 5.24] | 1.53 [0.67 - 3.48] | 1.31 [0.57 - 3.01] | 0.86 [0.46 - 1.60] |
| Insula | 1.01 [0.50, 2.04] | 1.24 [0.74, 2.07] | 1.92 [0.81 - 4.55] | 1.90 [0.87 - 4.17] | 1.55 [0.69 - 3.50] | 0.82 [0.44 - 1.53] |

*Note*. CD = conduct disorder only; TD = typically developing controls; mTBI = mild traumatic brain injury only; mTBI+CD = co-occurring mild traumatic brain injury and conduct disorder; OR = odds ratio; NAc = nucleus accumbens; ACC = anterior cingulate cortex; OFC = orbitofrontal cortex.

**Table S3.** Multinomial Regression Model Results Comparing Activation During Reward Anticipation Across Groups, Including IQ as a Covariate

|  | **Group comparisons** | | | | | |
| --- | --- | --- | --- | --- | --- | --- |
|  | **CD vs TD** | **mTBI vs TD** | **mTBI+CD vs TD** | **mTBI+CD vs CD** | **mTBI+CD vs mTBI** | **CD vs mTBI** |
| **ROI** | ***OR* [95% CI]** | ***OR* [95% CI]** | ***OR* [95% CI]** | ***OR* [95% CI]** | ***OR* [95% CI]** | ***OR* [95% CI]** |
| **Left hemisphere** |  |  |  |  |  |  |
| Amygdala | 1.13 [0.72, 1.80] | 1.37 [0.91, 2.06] | 1.02 [.60 - 1.73] | 0.90 [0.60 - 1.41] | 0.74 [0.46 - 1.20] | 0.83 [0.55 - 1.25] |
| NAc | 1.10 [0.70, 1.70] | 1.26 [0.90, 1.78] | 1.94 [1.11 - 3.41] | 1.78 [1.06 - 2.96] | 1.54 [0.91 - 2.59] | 0.87 [0.59 - 1.27] |
| Caudal ACC | 1.33 [0.76, 2.35] | 1.51 [0.97, 2.37] | 2.45 [1.26 - 4.79] | 1.84 [1.04 - 3.25] | 1.62 [0.87 - 3.03] | 0.88 [0.53 - 1.47] |
| Rostral ACC | 1.39[0.77, 2.51] | 1.36 [0.96, 1.93] | 2.31 [1.27 - 4.21] | 1.66 [0.93 - 2.98] | 1.69 [0.98 - 2.93] | 1.02 [0.61 - 1.70] |
| Medial OFC | 1.01 [0.78, 1.32] | 0.98 [0.82, 1.16] | 1.39 [1.00 - 1.94] | 1.37 [1.01 - 1.86] | 1.43 [1.04 - 1.95] | 1.04 [0.82 - 1.32] |
| Hippocampus | 1.13 [0.58, 2.18] | 1.47 [0.86, 2.52] | 1.18 [0.52 - 2.69] | 1.05 [0.50 - 2.23] | 0.80 [0.37 - 1.73] | 0.77 [0.42 - 1.39] |
| Thalamus | 1.49 [0.76, 2.93] | 1.72 [0.99, 2.97] | 2.04 [0.90 - 4.65] | 1.37 [0.64 - 2.91] | 1.19 [0.56 - 2.55] | 0.87 [0.48 - 1.58] |
| Insula | 1.24 [0.58, 2.66] | 1.41 [0.81, 2.46] | 1.98 [0.80 - 4.92] | 1.60 [0.70 - 3.65] | 1.40 [0.59 - 3.30] | 0.88 [0.44 - 1.75] |
|  |  |  |  |  |  |  |
| **Right hemisphere** |  |  |  |  |  |  |
| Amygdala | 1.08 [0.66, 1.78] | 1.33 [0.88, 2.02] | 1.03 [0.57 - 1.84] | 0.95 [0.57 - 1.60] | 0.77 [0.46 - 1.31] | 0.81 [0.53 - 1.25] |
| NAc | 1.07 [0.69, 1.66] | 1.14 [0.83, 1.56] | 1.83 [1.05 - 3.19] | 1.71 [1.02 - 2.87] | 1.60 [0.96 - 2.68] | 0.94 [0.64 - 1.38] |
| Caudal ACC | 1.12 [0.62, 2.01] | 1.13 [0.71, 1.78] | 1.79 [0.89 - 3.58] | 1.59 [0.83 - 3.05] | 1.59 [0.83 - 3.03] | 1.00 [0.59 - 1.68] |
| Rostral ACC | 1.16 [0.69, 1.96] | 1.04 [0.70, 1.53] | 2.14 [1.14 - 4.05] | 1.85 [1.01 - 3.39] | 2.07 [1.14 - 3.77] | 1.12 [0.70 - 1.79] |
| Medial OFC | 0.89 [0.69, 1.17] | 0.93 [0.77, 1.12] | 1.27 [0.88 - 1.82] | 1.42 [1.02 - 1.96] | 1.37 [0.97 - 1.92] | 0.97 [0.77 - 1.21] |
| Hippocampus | 0.94 [0.46, 1.92] | 1.59 [0.95, 2.68] | 1.14 [0.48 - 2.69] | 1.21 [0.54 - 2.71] | 0.71 [0.32 - 1.60] | 0.59 [0.31 - 1.10] |
| Thalamus | 1.41 [0.69, 2.85] | 1.63 [0.97, 2.76] | 2.15 [0.88 - 5.24] | 1.53 [0.67 - 3.48] | 1.31 [0.57 - 3.01] | 0.86 [0.46 - 1.60] |
| Insula | 1.01 [0.50, 2.04] | 1.24 [0.74, 2.07] | 1.92 [0.81 - 4.55] | 1.90 [0.87 - 4.17] | 1.55 [0.69 - 3.50] | 0.82 [0.44 - 1.53] |

*Note.* This model includes all of the original covariates (sex, ethnicity, age, ADHD, internalizing problems, low birth weight, premature birth, smoking or alcohol consumption during pregnancy, low parental education, low household income, and family conflict) as well as IQ. CD = conduct disorder only; TD = typically developing controls; mTBI = mild traumatic brain injury only; mTBI+CD = co-occurring mild traumatic brain injury and conduct disorder; OR = odds ratio; NAc = nucleus accumbens; ACC = anterior cingulate cortex; OFC = orbitofrontal cortex.

**Table S4.** Multinomial Regression Model Results Comparing Activation During Reward Receipt Across Groups, Including IQ as a Covariate

|  | **Group comparisons** | | | | | |
| --- | --- | --- | --- | --- | --- | --- |
|  | **CD vs TD** | **mTBI vs TD** | **mTBI+CD vs TD** | **mTBI+CD vs CD** | **mTBI+CD vs mTBI** | **CD vs mTBI** |
| **ROI** | ***OR* [95% CI]** | ***OR* [95% CI]** | ***OR* [95% CI]** | ***OR* [95% CI]** | ***OR* [95% CI]** | ***OR* [95% CI]** |
| **Left hemisphere** |  |  |  |  |  |  |
| Amygdala | 1.05 [0.70 - 1.56] | 0.87 [0.63 - 1.22] | 2.23 [1.32 - 3.78]* | 2.14 [1.30 - 3.52]* | 2.65 [1.53 - 4.29]* | 1.20 [0.81 - 1.77] |
| NAc | 1.28 [0.91 - 1.81] | 1.07 [0.81 - 1.43] | 1.57 [0.97 - 2.52] | 1.22 [0.79 - 1.89] | 1.46 [0.92 - 2.30] | 1.20 [0.87 - 1.63] |
| Caudal ACC | 1.38 [0.85 - 2.23] | 1.27 [0.86 - 1.89] | 2.49 [1.27 - 4.90] | 1.81 [0.97 - 3.36] | 1.96 [1.00 - 3.84] | 1.09 [0.68 - 1.73] |
| Rostral ACC | 1.38 [0.91 - 2.09] | 1.19 [0.84 - 1.69] | 1.72 [1.03 - 2.88] | 1.25 [0.78 - 2.00] | 1.44 [0.79 - 1.70] | 1.15 [0.88 - 2.36] |
| Medial OFC | 1.30 [1.02 - 1.65] | 1.20 [1.00 - 1.45] | 1.47 [1.10 - 1.98] | 1.14 [0.88 - 1.47] | 1.22 [0.93 - 1.61] | 1.08 [0.86 - 1.34] |
| Hippocampus | 1.64 [0.95 - 2.85] | 1.15 [0.73 - 1.80] | 4.24 [2.09 - 8.60]* | 2.58 [1.39 - 4.80]* | 3.69 [1.86 - 7.33]* | 1.43 [0.85 - 2.41] |
| Thalamus | 1.26 [0.72 - 2.22] | 1.12 [0.72 - 1.73] | 2.16 [0.91 - 5.13] | 1.71 [0.77 - 3.77] | 1.93 [0.83 - 4.49] | 1.13 [0.67 - 1.90] |
| Insula | 1.28 [0.71 - 2.29] | 1.08 [0.67 - 1.73] | 2.62 [1.22 - 5.62] | 2.05 [1.01 - 4.16] | 2.43 [1.18 - 5.02] | 1.19 [0.70 - 2.01] |
|  |  |  |  |  |  |  |
| **Right hemisphere** |  |  |  |  |  |  |
| Amygdala | 1.19 [0.81 - 1.76] | 0.88 [0.64 - 1.22] | 1.54 [0.94 - 2.52] | 1.29 [0.82 - 2.05] | 1.75 [1.07 - 2.88] | 1.35 [0.92 - 2.00] |
| NAc | 1.17 [0.82 - 1.65] | 0.90 [0.66 - 1.21] | 1.39 [0.82 - 2.36] | 1.19 [0.74 - 1.90] | 1.55 [0.92 - 2.61] | 1.30 [0.93 - 1.83] |
| Caudal ACC | 1.30 [0.79 - 2.12] | 1.10 [0.74 - 1.64] | 2.40 [1.29 - 4.48] | 1.85 [1.04 - 3.30] | 2.18 [1.18 - 4.01] | 1.18 [0.73 - 1.88] |
| Rostral ACC | 1.63 [1.07 - 2.47] | 1.36 [0.96 - 1.93] | 1.73 [1.05 - 2.86] | 1.07 [0.68 - 1.66] | 1.27 [0.78 - 2.08] | 1.19 [0.79 - 1.79] |
| Medial OFC | 1.35 [1.05 - 1.74] | 1.19 [0.98 - 1.46] | 1.49 [1.09 - 2.05] | 1.11 [0.86 - 1.43] | 1.25 [0.93 - 1.69] | 1.13 [0.90 - 1.42] |
| Hippocampus | 1.65 [0.91 - 3.01] | 1.28 [0.79 - 2.10] | 3.17 [1.57 - 6.38]* | 1.92 [1.13 - 3.27] | 2.47 [1.32 - 4.62]* | 1.29 [0.76 - 2.17] |
| Thalamus | 1.39 [0.83 - 2.33] | 1.35 [0.89 - 2.07] | 3.00 [1.45 - 6.18]* | 2.15 [1.10 - 4.22] | 2.21 [1.07 - 4.56] | 1.03 [0.62 - 1.69] |
| Insula | 1.10 [0.60 - 2.02] | 1.09 [0.69 - 1.73] | 1.63 [0.72 - 3.69] | 1.48 [0.70 - 3.11] | 1.49 [0.68 - 3.28] | 1.01 [0.58 - 1.76] |

*Note.* This model includes all of the original covariates (sex, ethnicity, age, ADHD, internalizing problems, low birth weight, premature birth, smoking or alcohol consumption during pregnancy, low parental education, low household income, and family conflict) as well as IQ. CD = conduct disorder only; TD = typically developing controls; mTBI = mild traumatic brain injury only; mTBI+CD = co-occurring mild traumatic brain injury and conduct disorder; OR = odds ratio; NAc = nucleus accumbens; ACC = anterior cingulate cortex; OFC = orbitofrontal cortex.

**p* <.05 (FDR-corrected)

**
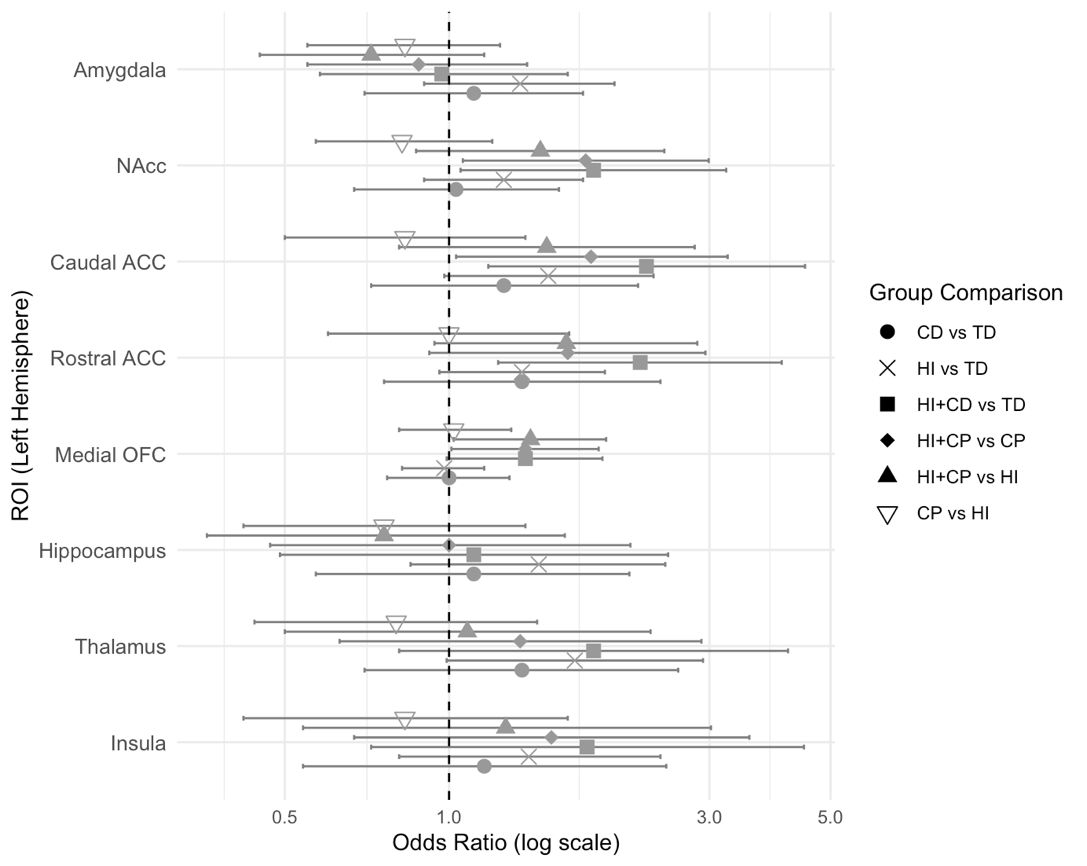

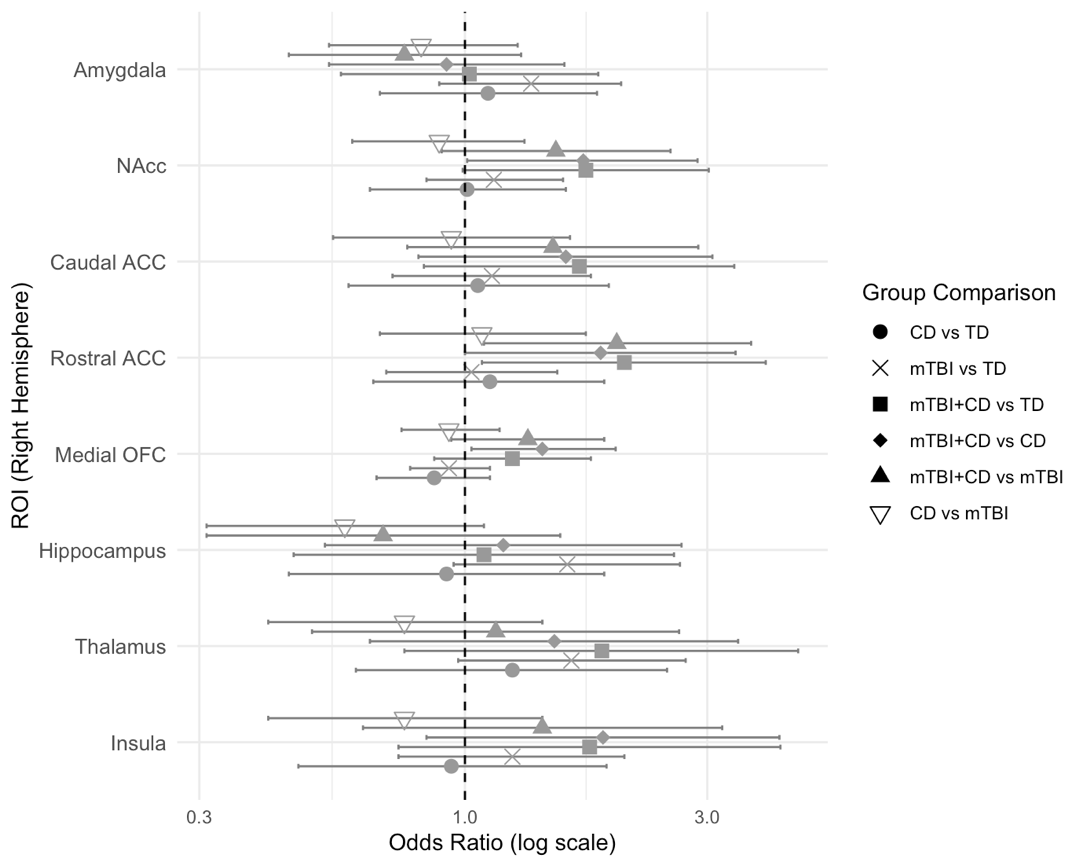
Figure S2.** A Figure of the Odds Ratios (and CI) for Reward Anticipation


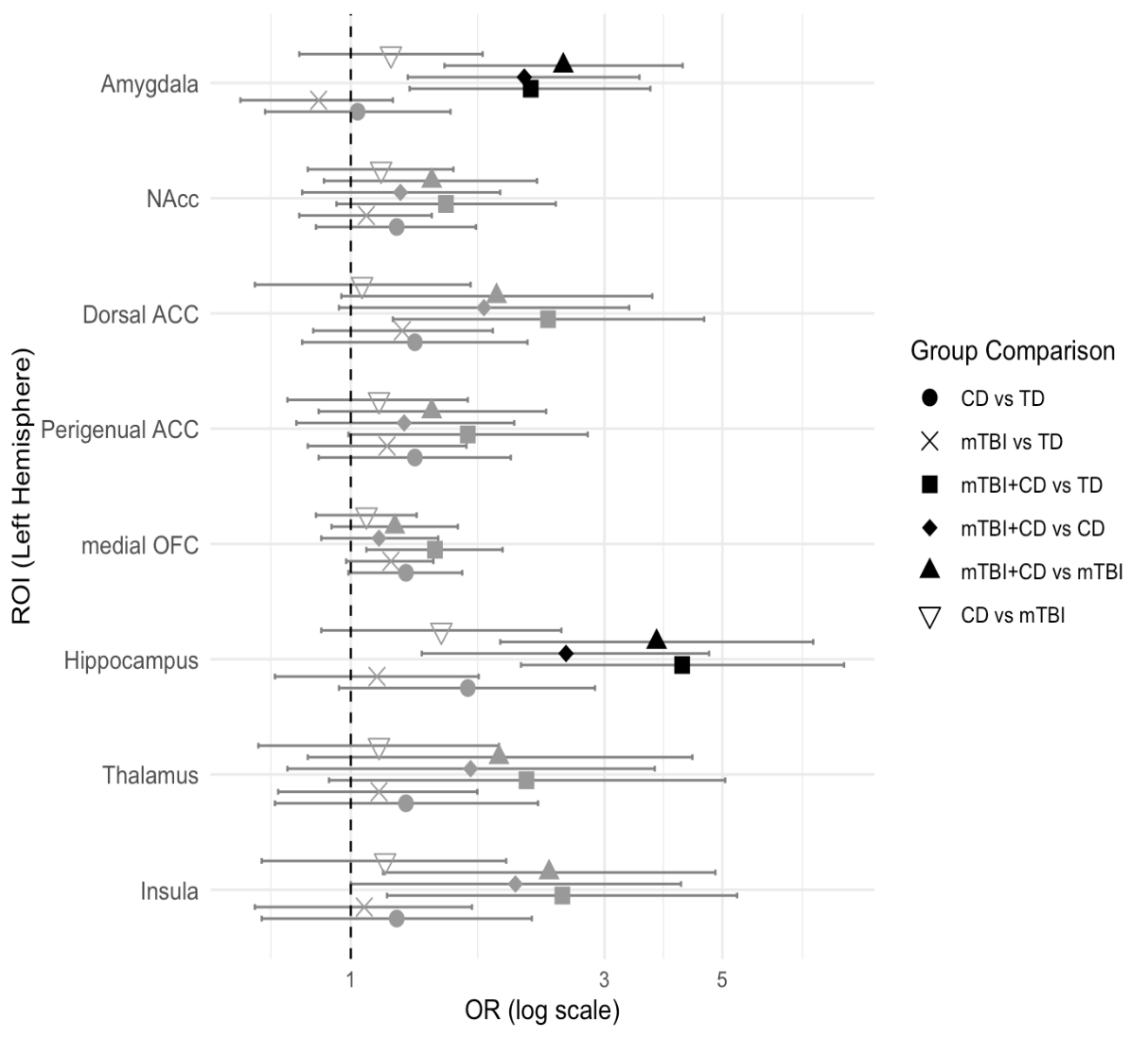


*Note.* This figure shows the odd ratios (ORs) and their confidence intervals during reward anticipation for the left and right hemispheres.

**
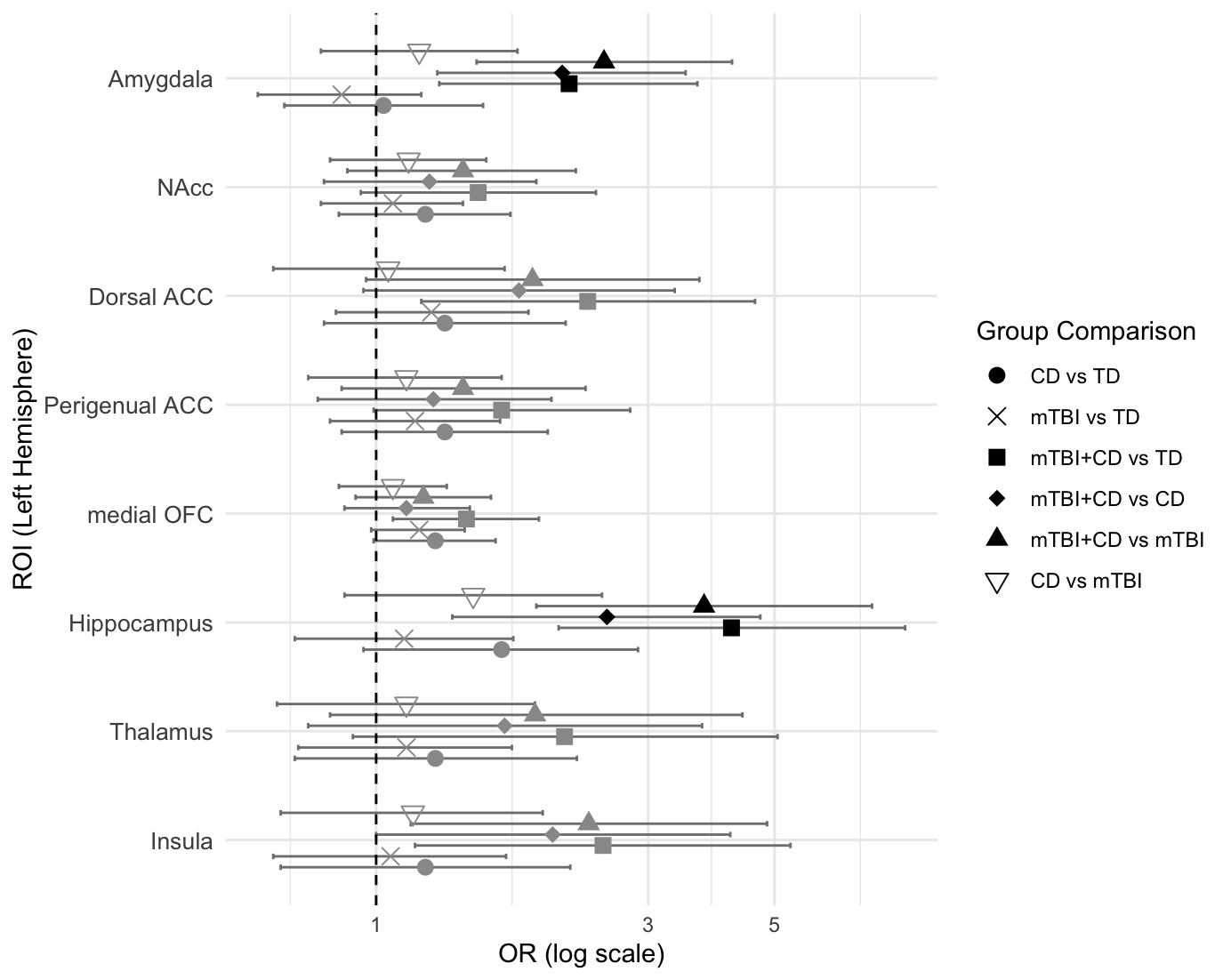

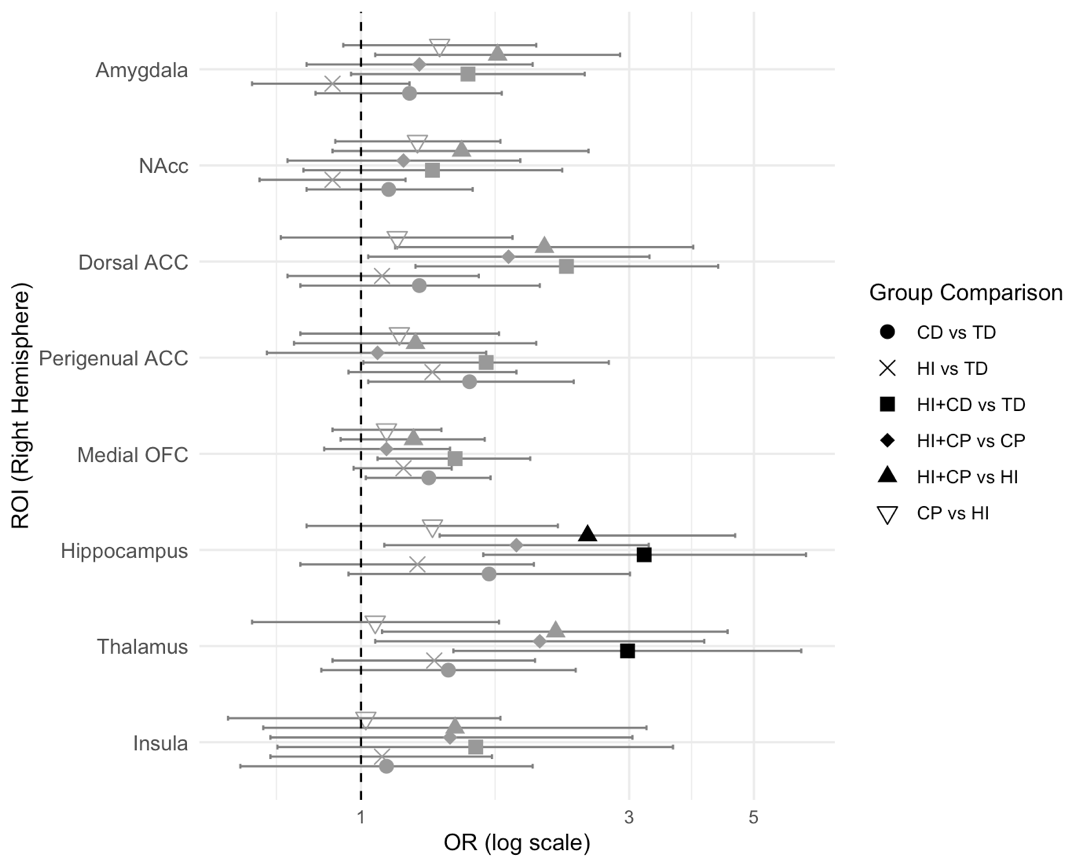

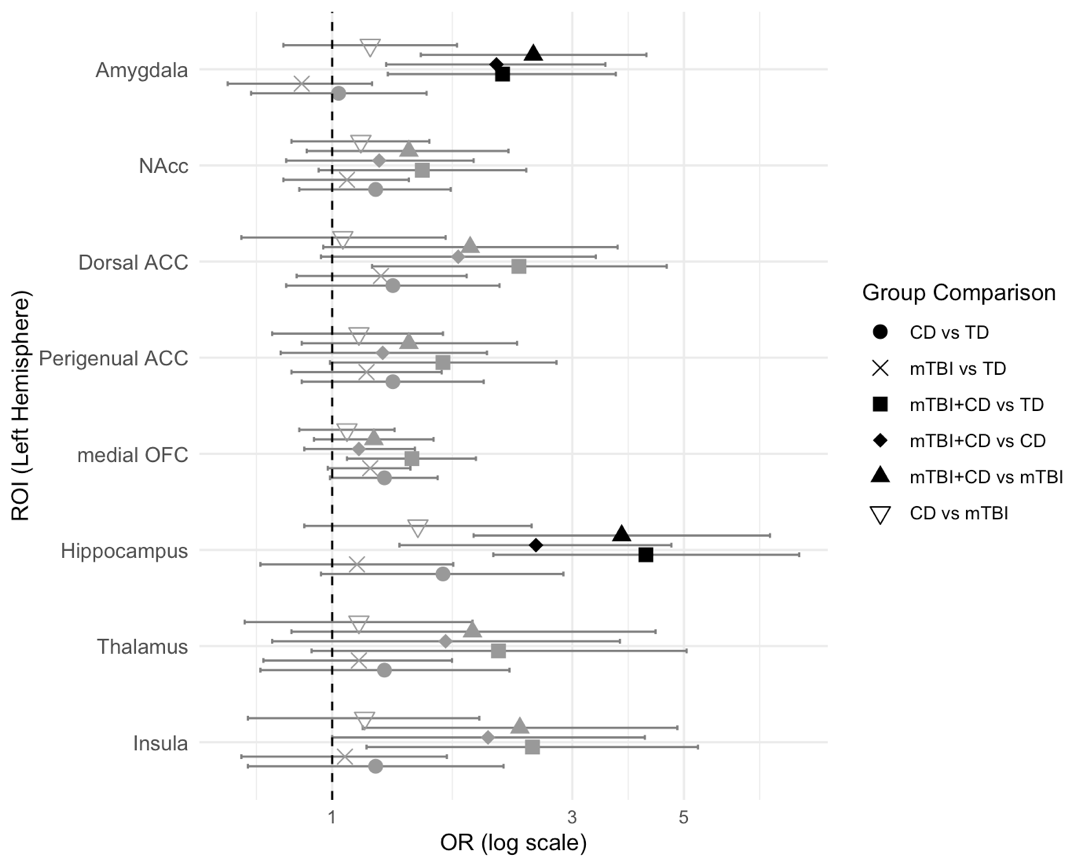
Figure S3.** A Figure of the Odds Ratios (and CI) for Reward Receipt

*Note.* This figure shows the odd ratios (ORs) and their confidence intervals during reward anticipation for the left and right hemispheres. Significant ORs (After FDR-correction) are in bold.

**Table S5.** Multinomial Regression Model Results Comparing Activation During Reward Receipt in the Left Amygdala and Hippocampus Across Groups, Excluding mTBI

|  | **Amygdala** | **Hippocampus** |
| --- | --- | --- |
| **Group Comparison** | ***OR* [95% CI]** | ***OR* [95% CI]** |
| mTBI vs TD | 2.16 [1.27 – 3.69] | 4.16 [2.06, 8.37] |
| mTBI+CD vs CD | 2.11 [1.27, 3.52] | 2.56 [1.38, 4.77] |
| mTBI+CD vs mTBI | 2.59 [1.52, 4.42] | 3.77 [1.91, 7.47] |

*Note.* CD = conduct disorder only; TD = typically developing controls; mTBI = mild traumatic brain injury only (improbable or possible TBI only); mTBI+CD = co-occurring mild traumatic brain injury and conduct disorder (improbable or possible TBI only); OR = odds ratio.

**p* <.05
